# A Study on the Behavior of a Polyurethane Drug Carrier in Various pH Media

**DOI:** 10.25122/jml-2020-0010

**Published:** 2020

**Authors:** Florin Borcan, Marius Mioc, Roxana Ghiulai, Iulia Pinzaru, Cristina Adriana Dehelean, Codruta Marinela Soica

**Affiliations:** “Victor Babes” University of Medicine and Pharmacy Timisoara, Faculty of Pharmacy, Timisoara, Romania

**Keywords:** Aggregation, drug delivery system, isocyanate, polymer, Zetasizer, silver-based nanoparticles (AgNPs), 1,4-butanediol (BD), 1,6-hexamethylene-diisocyanate (HMDI), isophorone-diisocyanate (IPDI), mono-ethylene glycol (MEG), polydispersity index (PDI), polyethylene glycol (PEG), polyurethane (PU)

## Abstract

Polyurethane nano- and micro-structures have been studied intensively in the last decade as drug delivery systems for various herbal extracts as well as pure active biological substances. Their biocompatibility, haemocompatibility, safe degradation, and low-cost production are just a few advantages of these materials that were already used in numerous medical applications (catheters, surgical drapes, wound dressing). The primary purposes of this study include obtaining empty polyurethane microstructures and the assessment of their modifications in media with different pH values. A mixture of two aliphatic diisocyanates and an aqueous phase based on a polyether were used during the synthesis process. The size, homogeneity, and surface charge were studied using a Cordouan Technol. Zetasizer, while the pH measurements were conducted with a portable pH Meter Checker®, Hanna Instruments. The results showed the obtaining of an almost homogeneous sample containing microstructures with sizes ranging between 139 and 151 nm, with a pH value of approximately 6.78 and a Zeta potential of 24.6. Expected decreases in microparticles’ sizes were observed in all types of media during a 15-days experiment, but the process was accelerated by a low pH when an increase of the Zeta potential value was noticed as well. Our data provide new information about the degradation process of the polyurethane microstructures on the one hand and the drug release rate of these materials when used as drug carriers, on the other hand.

## Introduction

The oral route is the most frequently used in drug administration [1], as liquid and solid formulations. The effect of oral drugs can be local, when the active substance induces ulcer healing, stimulates or inhibits the peristaltic intestinal movement (purgatives and spasmolytics, respectively) or alters the digestive processes in the stomach and intestines; a general effect occurs when the drugs are absorbed through the gastrointestinal wall, penetrate the bloodstream and carry out their biological activity at a systemic level [2,3].

Unfortunately, although it is a convenient pathway, which offers the advantage of self-management, without any risk of infection, the oral route exhibits a few disadvantages: a slow rate of the therapeutic effect, the impossibility to administer certain drugs due to their low gastrointestinal absorption or high gastrointestinal side effects, the effect of first liver passage [4]. Also, the oral route cannot be used in comatose patients, during seizures or in case of severe lesions of the oral cavity [5].

The drug delivery systems, also known as drug carriers, are used when existing pharmaceutical formulations do not provide the necessary qualities for a specific medical purpose. These organic or inorganic nano- and micro-structures may encapsulate the active drugs in order to modify their physicochemical properties and to increase drug absorption; by adequately selecting the delivery system, one can increase the therapeutic effect of the encapsulated drug or can ensure its prolonged or targeted release, thus avoiding the risks of under- or overdose. An essential factor in terms of drug release from some carriers is the pH value of the biological environment. Y. Yao et al. [6] described the preparation of some biodegradable pH-sensitive polyurethane (PU) micelles based on polyethylene glycol (PEG), which were used as carriers for the anti-cancer drug paclitaxel. They modified the polyurethane chains by using PEGylated diethanolamine as a chain extender, and the results indicated an increased cytotoxic effect against the H460 lung cancer cells compared to other PEGylated polyurethane micelles that were used as a reference. Therefore, the authors concluded that the structure of the polyurethane carrier significantly influences the loading and pH-triggered release as well as the intracellular delivery of paclitaxel.

This study aimed to prepare polyurethane microstructures with a mean diameter ranging between 100 and 150 nm and to assess their behavior in media with different pH values.

## Material and Methods

### The reagents

1,4-butanediol (BD) was obtained from Carl Roth GmbH (Karlsruhe, Germany), while 1,6-hexamethylene-diisocyanate and isophorone-diisocyanate (HMDI and IPDI), polyethylene glycol, M≈200 (PEG), and Tween® 20 from Merck (Darmstadt, Germany). Mono-ethylene glycol (MEG) was purchased from Lach-Ner s.r.o. (Neratovice, Czech Republic). The substances that were necessary to obtain media with different pH values (phosphates, Glycine, HCl and NaOH, analytical grade purity) were obtained from SC Chimopar Trading SRL (Bucharest, Romania). All reagents were used without any previous purification.

### The synthesis protocol

**Figure 1:**
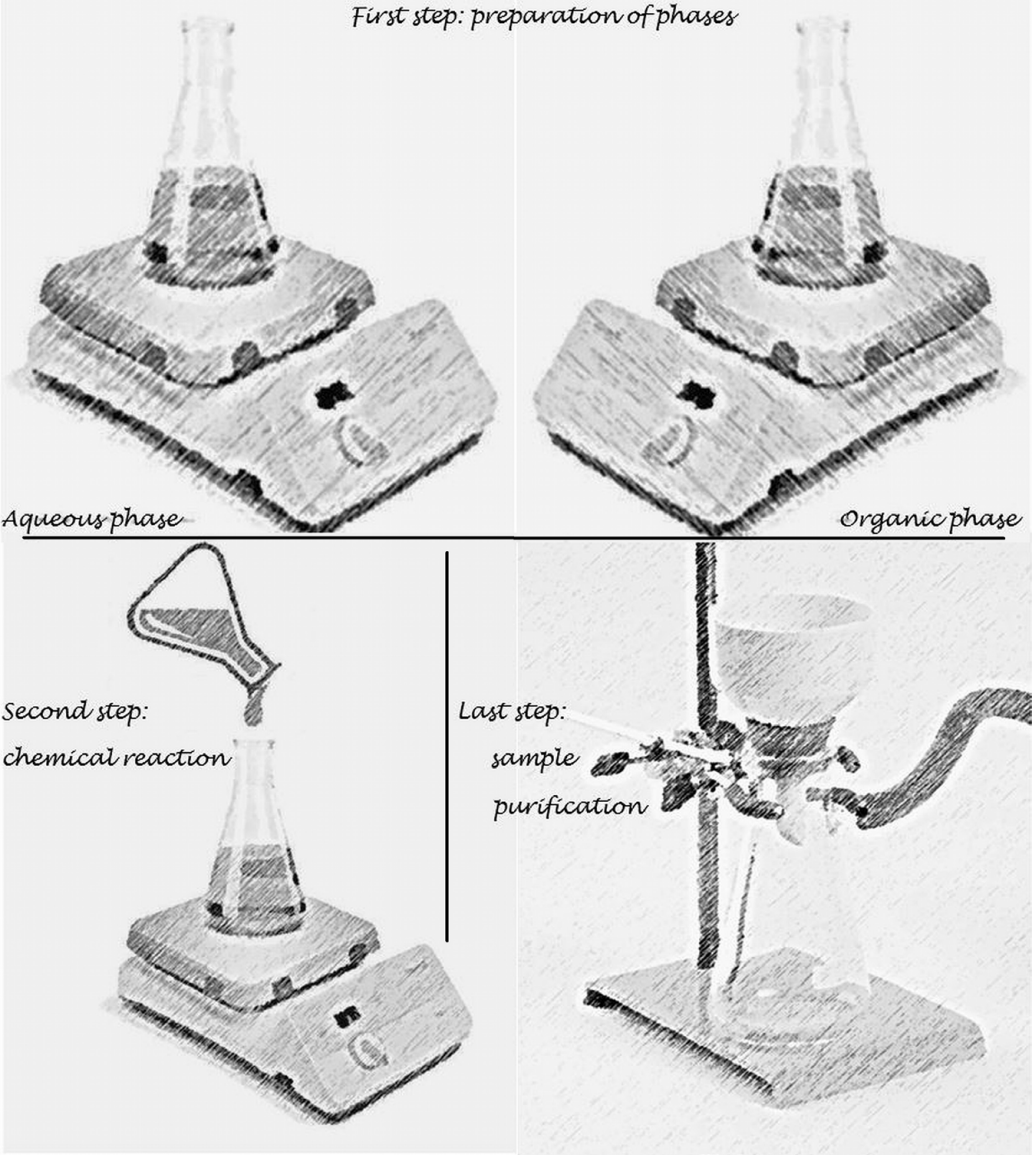
The multi-step procedure for the PU microstructures.

The obtaining of the PU carrier is based on a procedure with a few important steps that were already described in our previous papers [7-9] (Figure 1):

1. The preparation of the two phases:
  • an organic, non-aqueous phase based on a mixture of isocyanates (1.4 ml HMDI and 2.1 ml IPDI) and 1.0 ml Tween® 20 dissolved in 30 ml of acetone under magnetic stirring at around 400 rpm and 35° for 10 minutes;
  • an aqueous phase containing 3.0 ml BD and 5.0 ml PEG in 45 ml distilled water, homogenized at 400 rpm, and 35° for 10 minutes.
2. The obtaining of PU structures:
  • the phases were rapidly mixed under magnetic stirring at around 550 rpm, at 20°; the stirring continued for 200 min to ensure the complete formation of PU macromolecular chains.
3. The purification of the synthesized product:
  • the obtained suspension was repeatedly washed with a water-acetone mixture (1:1.4, v/v) using a glass Buchner funnel with a sintered disc, G4, 200 mL in order to homogenize the capsules size and to remove any trace of unreacted raw materials. Finally, the sample was dried as a thin layer in Petri dishes at room temperature until no mass change was observed (around 4-6 days).

### The characterization of the sample

The first evaluation of the synthesized sample was the measurement of PU microstructures pH. A portable pH Meter Checker® - Hanna Instruments (Woonsocket, RI, USA) was used according to the protocol already described by our team [10]: briefly, the device was first calibrated using Mettler Toledo (Schwerzenbach, Switzerland) standardized technical buffer solutions with pH=4.01 and 7.00, respectively, with a ± 0.02 standard error, at 25°. The pH probe was repeatedly washed with distilled water, and a diluted aqueous suspension of PU carrier (0.8 mg/ml) was used for the measurement; the pH was determined in triplicate.

A professional Zetasizer module Cordouan Technol. (Pessac, France) containing a Vasco Particle Size Analyzer and a Wallis Zeta potential Analyzer was used to determine the PU microstructures size and Zeta potential values, which can be used to predict their tendency to form clusters. The following parameters were set for the determinations: measurement temperature (22 ± 1°), the time interval (16 ± 3 μs), number of channels (460 ± 20), power of the laser (85 ± 5 %), acquisition mode (continuous), analysis mode: Pade-Laplace, a medium resolution for Wallis Analyzer, and Smoluchowski model as Henry function. The same 0.8 mg/ml suspension was used for these determinations.

The monitoring of the structures’ behavior in media with different pH is based on the size and Zeta potential measurements before and after the maintaining of little amounts (3.5 ml suspension with the same concentration, 0.8 mg/ml) of obtained product in prepared media with different pH values: 3.0 (obtained with a buffer solution of Glycine-HCl, 0.1 M), 7.0 (phosphate buffer, 0.1 M) and 10.0 (Glycine-NaOH, 0.1 M). Samples of 1.5 ml from every buffer solution were extracted every second day (at the same hour), and they were replaced with the same amount of fresh buffer solutions. The samples were inserted in polystyrene cuvettes for Zeta potential measurements; 3-5 drops were used to determine the size changes.

## Results

The initial determinations reveal the obtaining of a colloidal suspension containing PU microstructures described by a single-population with a gaussian distribution of diameters between 139 and 151 nm and high homogeneity (the polydispersity index, PDI=0.2). The pH value of this suspension was 6.78 ± 0.13, while the Zeta potential was 24.6 ± 1.2.

Table 1 describes the changes in the size and Zeta potential of PU microstructures during a 15-days experiment.

**Table 1:** The evolution of size and Zeta potential of PU microstructures.

**Period, days**	**Measured values at. different pH**					
	**3.0**		**7.0**		**10.0**	
	**Mean size, nm**	**Zeta potential, mV**	**Mean size, nm**	**Zeta potential, mV**	**Mean size, nm**	**Zeta potential, mV**
**1**	144	24.5	147	24.8	142	24.7
**3**	138	24.6	143	24.5	139	24.1
**5**	131	24.8	131	24.6	130	23.9
**7**	126	25.1	127	24.7	122	23.5
**9**	118	25.4	125	24.8	119	23.3
**11**	117	25.5	125	24.9	120	22.9
**13**	111	26.4	123	24.3	118	22.0
**15**	108	27.1	122	24.0	117	20.7

## Discussion

According to Albulescu et al., the size evolution in time of structures used as drug delivery systems can be used as a prediction parameter for the drug release rate [11], which is presumably the main parameter in the assessment of a drug carrier. It is known that a controlled release system allows the introduction of a drug into the body with improved effectiveness and safety by controlling the rate, the time interval, and the place of drug release inside the body [12]. During the current research, a 25.0% and 17.6% decrease in particle diameter were recorded within an acidic and basic environment, respectively. A 17.0 % decrease of PU microstructures diameter was found in the media with a neutral pH. The decrease of the microstructures’ size is a normal process due to their degradation process; a carrier that does not degrade over a specific time interval cannot be used as a drug delivery system. The accelerated degradation process in the acidic media can be correlated with the increasing of Zeta potential values that reveal higher stability against particle aggregation and, subsequently, more effective separation of the individual microparticles.

Zeta potential measurements are often used to predict the tendency of colloidal solutions or suspensions to agglomerate into clusters. Salopek et al. [13] reported that nano- and/or micro-structures with a Zeta potential within the range of 20-30 mV have a medium stability degree against the tendency to aggregate; the most unstable systems show Zeta potential values between -10 and +10 mV.

The pH value of the liquid environment has various influences on nanoparticle size and behavior. In contrast, silver nanoparticles (AgNPs) exhibit smaller sizes at higher pH values, resulting in lower aggregation and increased stability [14]; polymer particles containing polylactic acid and polyethylene glycol moieties show larger sizes and lower Zeta potential values in the same pH range. [15]. In the current study, the newly synthesized microstructures revealed a 10.6% increase in the Zeta potential within an acidic medium, an almost equal trend in a neutral pH medium, and a 16.2% decrease in an alkaline medium (pH = 10). These changes are in line with previously published reports in the field [16-18].

Tamer and Chen have reported the preparation of a lysine-based biodegradable poly (beta-aminoester urethane) network that can be used for local drug delivery [19]. The material is pH-sensitive, and a non-Fickian mechanism describes its water diffusion. The versatility of polyurethane materials was proven in different industries decades ago, and lately, it was used to produce a wide range of medical applications. This high versatility of PU is based on their biocompatibility as well as on the easy and low-cost possibility to modulate their elasticity, toughness, degradation period, and others.

The use of cross-linking agents with different functionality mixed with polyols that are the primary raw materials of the aqueous phase leads to the preparation of PU materials with hydrophilic surfaces that can enhance the osteogenic differentiation [20]. This modification of the hydrophilic character improves the aqueous solubilization of PU drug carriers and leads to a more intense therapeutic effect of active substances with low water solubility.

Nowadays, copolymers are often preferred to pure PU. Various systems based on two isocyanates (an aliphatic one - hexamethylene diisocyanate, and an aromatic one - 4,4’-diphenylmethane diisocyanate) coupled with bis-1,4-(hydroxyethyl) piperazine, N-methyldiethanolamine, and N-butyldiethanolamine (BDEA), respectively, were developed for doxorubicin delivery by Wang et al. [21]. Although the use of aromatic isocyanates leads to improved mechanical properties and this research group found that the final materials are non-toxic based on cytotoxicity evaluations, we have chosen to synthesize a PU carrier based on non-carcinogenic raw materials (aliphatic isocyanates) based on the information from other studies.

## Conclusions

Polyurethane nano- and micro-structures represent a relatively new class of delivery systems in the pharmaceutical field. This paper describes a multi-step synthesis which combines a polyaddition reaction with spontaneous emulsification. Micro-structures with diameters around 145 nm, with a medium tendency to aggregate, a polydispersity index of 0.2, and an almost neutral pH in aqueous solution were obtained. The structures were assessed in three media with different pH values, and an accelerated decrease of their size was reported in an acidic medium; this behavior can be correlated with the increase of Zeta potential values, which indicates higher stability against the tendency to form clusters. Moreover, the accelerated degradation of polyether-urethanes in acidic medium indicates their potential to be used in oral drug administration.

## Conflict of Interest

The authors declare that there is no conflict of interest.
